# A Telehealth-Adapted Dementia Caregiver Skills Training Intervention (TeleCARE): Single-Arm Pre-Post Intervention Study

**DOI:** 10.2196/81256

**Published:** 2026-03-12

**Authors:** Maureen K O'Connor, Steven D Shirk, Jaye E McLaren, Andrew H Nguyen, Kendra Pugh, Madeline A Sullivan, Emily E Metcalf, Samantha Harrington, Lauren R Moo

**Affiliations:** 1 Department of Neuropsychology, Edith Nourse Rogers Memorial Veterans Hospital, Bedford, MA, United States; 2 Department of Neurology, Chobanian & Avedisian School of Medicine, Boston University, Boston, MA, United States; 3 Geriatric Research Education and Clinical Center, Edith Nourse Rogers Memorial Veterans Hospital, 200 Springs Road, Bedford, MA, 01730, United States, 1 781-687-2830; 4 Department of Psychiatry and Behavioral Sciences, Department of Population and Quantitative Health Sciences, University of Massachusetts Chan Medical School, Worcester, MA, United States; 5 Mental Illness Research Education and Clinical Center, Edith Nourse Rogers Memorial Veterans Hospital, Bedford, MA, United States; 6 Department of Neurology, Harvard Medical School, Boston, MA, United States

**Keywords:** Alzheimer disease, caregiver education, dementia, older adults, telehealth, caregivers, telemedicine, veterans, neuropsychiatric symptoms

## Abstract

**Background:**

Dementia caregivers often want to support aging at home, but as neuropsychiatric symptoms (NPS) become more severe, caregiver challenges increase, often resulting in negative outcomes for both the caregiver and care recipient and institutionalization. Project CARE is a manualized in-person group intervention for dementia caregivers designed to reduce negative caregiver outcomes by teaching skills to manage NPS in care recipients in the home environment. Interventions that occur in person, however, can be difficult for caregivers to attend. Telehealth-based interventions are possible alternatives that reduce barriers to attendance.

**Objective:**

The primary objective of this pilot study was to evaluate the feasibility and acceptability of offering CARE via telehealth (TeleCARE). The secondary objective was to explore quantitative outcome trends and effect sizes postintervention outcomes of TeleCARE for both caregivers and care recipients.

**Methods:**

Rates of recruitment, attendance, and completion were used to assess the feasibility of TeleCARE. Data on technology use and telehealth-based adaptations were also collected. Acceptability was measured using participants’ rated satisfaction with the intervention immediately postintervention. Questionnaires were administered at baseline and immediately and 3 months postintervention. Primary outcomes for exploratory analysis included NPS presence, severity, and caregiver NPS-related distress. Secondary outcomes included caregiver depression, anxiety, stress, self-efficacy, positive aspects of caregiving, and meaning and purpose in life.

**Results:**

Of the 109 caregivers contacted for recruitment, 24 (22%) caregivers enrolled in TeleCARE, and 20 (83%) caregivers, predominantly female spouses, completed the study. Feedback from participants in the TeleCARE test group 1 (n=3) was used to modify the intervention to improve the telehealth experience, including adding procedures to improve safety, encourage rapport building, address etiquette, and ensure privacy. The final version of TeleCARE included 7 weekly synchronous video sessions. Ten out of 17 participants (59%) attended all 7 sessions, and all participants attended at least 5 sessions. Satisfaction ratings suggested adequate intervention acceptability. Most participants (11/17, 65%) required technological support, which was needed throughout the intervention. Quantitative trends were observed toward postintervention decreases in care-recipient NPS severity (Cohen *d*=0.16), caregiver depression (*d*=0.15), anxiety (*d*=0.23), and caregiver self-efficacy (*d*=0.21), as well as increases in positive aspects of caregiving (*d*=0.18) and meaning and purpose in life (*d*=0.09). Most improvements were not sustained at the 3-month follow-up.

**Conclusions:**

In this pilot feasibility study, dementia caregivers were successfully recruited and engaged in TeleCARE. Overall, TeleCARE was deemed feasible and acceptable. The current findings suggest that offering interventions via telehealth requires modifications and technological support for older caregiver engagement but is a feasible and acceptable means of offering services.

## Introduction

Most individuals with dementia experience neuropsychiatric symptoms (NPS), changes in emotional expression and behavior, during the disease course [1-3]. The presence of NPS is associated with increased caregiver depression and burden and decreased quality of life for both the caregivers and care recipients [4]. Although most dementia caregivers want to keep the care recipient aging at home [5], the presence of NPS often spurs institutionalization [6-9]. The association between NPS and institutionalization has been shown to be mediated by caregiver burden and distress [8]. Interventions that teach dementia caregivers skills to manage NPS have the potential to reduce negative caregiver and care recipient outcomes and institutionalization of the care recipient [6,9,10]. However, due to caregiving demands, attending in-person interventions can be prohibitive. Telehealth offers a solution.

The resources for enhancing Alzheimer’s caregiver health (REACH) study [11] is one of the most well-studied, targeted, skills-based interventions aimed at improving caregiver well-being. Participation in REACH decreases dementia caregiver anxiety, depression, and burden [12-14]. The results of REACH suggest that targeted skills-based training may be more effective than broader psychoeducational interventions [11]. CARE was designed as a briefer intervention compared with REACH. CARE is a 5-session group intervention for dementia caregivers that teaches skills to manage NPS in the home environment through behavioral management training, pleasant events scheduling, and emotional regulation techniques [15]. CARE sessions run for 90 minutes weekly and are highly structured, consisting of primarily didactic learning and practice, with some space for content-related open discussion. CARE has been shown to reduce caregiver burden related to NPS. REACH, CARE, and other similar programs demonstrate that providing dementia caregivers with specific skills training to manage NPS can be an effective way to support caregivers and improve caregiver outcomes. Unfortunately, many dementia caregiver skills training programs have historically been offered in person only, limiting accessibility for people with barriers to accessing in-person services.

Many dementia caregivers have unique barriers to in-person care. For example, in 2025, 60% of dementia caregivers were working an average 35 hours per week while caregiving [5], limiting the time available to travel to and participate in interventions. Dementia caregivers also have disproportionate financial stressors, incurring approximately 4 times the average out-of-pocket costs compared with nondementia caregivers, creating a financial barrier to using in-person services (eg, they cannot pay for gas, respite care, or take time off from work) [5]. Other dementia caregiver–specific concerns that limit in-person accessibility of services include feeling uncomfortable leaving the diagnosed individual at home and having difficulty finding respite care [16,17]. For caregivers living in rural areas, there is less awareness of available services, greater difficulties accessing transportation, and barriers related to the sheer distance needed to travel for services compared to urban caregivers [18,19]. We can address these challenges by offering interventions via video telehealth [20].

The acceptance and use of telehealth in dementia care has expanded in recent years, demonstrating positive impacts on depression, anxiety, loneliness, NPS management, and satisfaction and quality of life for both caregivers and care recipients [21]. Rapid expansion of telehealth during the COVID-19 pandemic changed the landscape of the health care system [22]. Older adults and their caregivers expressed satisfaction with telehealth and a desire to keep using telehealth postpandemic [23]. Studies of dementia caregiver telehealth support groups have demonstrated effectiveness in improving caregiver burden, reducing depressive symptoms, and increasing coping skills [24-27]. A systematic review [28] suggested that telehealth supportive interventions were potentially as effective as in-person interventions for reducing dementia caregivers’ depression, anxiety, stress, and improving self-efficacy. One recent meta-analysis and systematic review [29] specifically investigating the effectiveness of telehealth skills training for management of NPS in dementia found improvements in caregivers’ ability to manage NPS, quality of life, and stress levels.

The primary goal of this study was to evaluate the feasibility and acceptability of offering CARE via a telehealth format (TeleCARE) to reduce many of the current barriers that dementia caregivers face in accessing skills training to manage NPS. Keeping the small sample size of this pilot study in mind, the second objective was to explore quantitative outcomes of TeleCARE with trends and effect sizes.

## Methods

### Participants

We recruited participants from the Bedford, Massachusetts, and Manchester, New Hampshire, Department of Veterans Affairs Medical Centers and surrounding communities using posted informational flyers. We also reviewed Veterans Affairs (VA) medical records to identify veterans with dementia and mailed recruitment letters to their homes and followed up with phone calls. Criteria for study inclusion were (1) providing informal care for someone with self-reported or physician-confirmed diagnosis of dementia, (2) at least 1 caregiver-reported NPS for the care recipient at the time of enrollment, (3) provision of a minimum of 5 hours per week of direct caregiving, and (4) access to a computer or tablet and internet or Wi-Fi. Participants could be caregivers of veterans with dementia or veterans caring for someone with dementia.

Pilot and feasibility work typically aims to explore recruitment, engagement, and practicality rather than statistical power [30,31]. Therefore, for this pilot study, the focus was on testing feasibility and acceptability rather than testing hypothesis-driven significance with associated power calculations for sample size. To accomplish these goals, we selected a target enrollment of 24 caregivers.

### Ethical Considerations

This study was approved and monitored by the VA Bedford Health Care System Institutional Review Board (study no. 1598706). Written informed consent was obtained from all study participants. Privacy and confidentiality of all research participants’ data and identities were maintained using multiple methods, including using a Health Insurance Portability and Accountability Act–compliant web-based platform with end-to-end encryption, coding data so that only the research team had access to identifiable information, storing data securely behind the VA firewall and restricting access to the study team, analyzing coded data and reporting data in aggregate form, and establishing ground rules during group sessions to help participants protect their privacy. Participants received US $25 for completing each of the baseline and postintervention follow-up visits, and US $50 for completing the 3-month follow-up visit, receiving a possible total of US $100 to compensate for their time.

### Methodological Adaptations, Procedures, and Intervention Description

Initially, we intended to conduct preintervention, in-person sessions to obtain written consent and complete baseline assessments, as well as to provide in-person instruction about technology use. However, prior to the start of the study, the COVID-19 pandemic forced us to convert all study visits to a virtual format. We added a preintervention live video session to provide technology instruction before the start of groups. Individual assistance to help participants access the initial tutorial was provided as needed by phone. Follow-up data collection visits were conducted by video immediately and 3 months post-treatment. Participants were compensated for their time.

A trained interviewer met with each eligible caregiver via video to explain the study, answer questions, review the Health Insurance Portability and Accountability Act authorization, and sign the informed consent forms. The interviewer also provided initial training on using the videoconferencing platform. During the same visit or a second visit, depending on participant preference, the interviewer administered the baseline questionnaires. Questions were presented visually via PowerPoint (Microsoft Corp) one at a time and read aloud. Participant responses were recorded in VA REDCap (Research Electronic Data Capture), a secure data management software, in real-time. After baseline assessment, a printed TeleCARE manual was mailed to the participant. All caregivers began the intervention within 6 weeks of baseline assessment. The collection of immediate and 3-month follow-up data followed the same procedures as baseline data collection. Additional supplemental visits took place by video or phone as needed to help participants troubleshoot technology issues. All video visits were conducted via Health Insurance Portability and Accountability Act–compliant, encrypted, VA-approved videoconferencing platforms.

TeleCARE was adapted from the original manualized CARE intervention [15]. As in CARE, TeleCARE groups met weekly for 90-minute sessions. At the beginning of each session, a doctoral-level occupational therapist leading the groups went through a series of prompts specific to telehealth to ensure safety and confidentiality during the session. Participants were given time during each session to discuss homework, troubleshoot any problems that may have arisen when attempting to use new skills, and offer each other support and advice.

The intervention was delivered to caregivers in 6 groups that ran sequentially from June 2021 to September 2022. We started with an initial TeleCARE test group (TeleCARE-test) to troubleshoot any issues before the formal pilot. TeleCARE-test participants reported that the 5 sessions did not provide adequate time to cover all the intervention material and allow for discussion. In response, we reviewed all intervention sessions to determine the number of additional sessions needed. The finalized program was extended to 7 sessions. Table 1 provides a summary of each session included in the final manualized intervention. Other intervention-specific modifications to adapt the program to the telehealth setting were made based on the experiences of the study team and feedback from the TeleCARE-test group. At the beginning of each session, we added unique steps to ensure participant safety in the telehealth setting. These steps included gathering information about where the participant was located in the event of a psychological (eg, expressed suicidal intent) or medical (eg, cardiac arrest) emergency. Unlike in-person interventions, telehealth interventions require the provider to know where the participant is located if the need arises to alert emergency services (eg, local police). The group leader also asked for updated contact information at the start of each session for participants joining from a new, unknown location. This information was provided to the group leader through secure chat to maintain privacy. The interventionist encouraged all participants to sign into the platform with their first name to facilitate group rapport. It became clear that video-specific etiquette guidelines should be added to the first session, including muting oneself when not speaking, using the raise hand feature, remaining primarily stationary during the group (eg, not walking around with the video device), and reducing background distractors (eg, television, pets, children). Participants were also asked to locate themselves in a suitable space to reduce privacy concerns (ie, a location where they could not be overheard discussing personal information). Finally, the need for broad technological support for older adults enrolled in TeleCARE. In response, we added a technology tutorial session before the intervention started, as noted above, and access to ongoing support as needed.

**Table 1.** Telehealth adaptation of CARE (TeleCARE) group sessions.

Session	Description
1‐2	*Introduction*: Caregivers introduce themselves to begin building group cohesion. Facilitators review dementia and caregiver burden, with a focus on neuropsychiatric symptoms, the importance of increasing pleasant activities as one method for improving care recipients’ behavior, and the caregiver–care recipient relationship. Facilitators present a Pleasant Activity Log.
3	*Increasing Pleasant Activities and Improving Communication*: Discuss the importance of engaging in pleasant activities, suggest common pleasant activities and strategies for generating personally meaningful pleasant activities, and provide education about how to increase pleasant activity engagement. Introduce strategies to improve communication. *Caregiver Homework*: Engage in one new pleasant activity daily and record it, generate additional ideas for pleasant activities, and compliment the care recipient at least once per day.
4‐5	*Increasing Pleasant Activities and Understanding Behavior*: Review homework and problem-solve any difficulties with homework. Review strategies for increasing pleasant activities. Introduce behavioral management theory and teach participants to identify antecedents, behavior, and consequences (ABCs) to assist with behavior management. Discuss triggers for problem behaviors and how they can be avoided. *Caregiver Homework*: Engage in one pleasant activity daily. Identify and record 3 patient problem behaviors.
6	*Understanding and Changing Difficult Behaviors*: Review homework and problem-solve any difficulties with homework. Practice goal setting and methods for changing problem behaviors in the home. *Caregiver Homework*: Engage in 2 pleasant activities daily. Choose one difficult behavior and attempt to modify it.
7	*Final Review*: Review homework and problem-solve difficulties caregivers had while modifying behaviors at home. Provide guidance on how to continue using these new skills in the future. Caregivers discuss and explore plans for future use of strategies. Celebrate completion of the group, participant contributions, personal growth, challenges, and willingness to participate.

### Measures

Following CONSORT (Consolidated Standards of Reporting Trials) guidelines [32] and the proposed guidelines for reporting feasibility study outcomes [33], we collected multiple measures of study feasibility, including recruitment rates, session attendance rates, study completion rates, and a formal rating of the acceptability of the intervention. Given our interest in the telehealth adaptation, we also collected data on technology use. Participants completed outcome measures at baseline, 1 to 2 weeks immediately after the intervention, and 3 months postintervention.

At baseline, caregivers completed a demographic and historical information survey, providing information about themselves and the care recipient. Caregivers also completed [1] the clinical dementia rating sum of boxes [34], a 5-point interview that characterizes 6 domains of the care recipient’s cognitive and functional performance and disease severity.

At baseline, immediate follow-up, and 3-month follow-up, caregivers were asked to report on the presence of the care recipient’s NPS and functional abilities. The Lawton Instrumental Activities of Daily Living (IADL) Scale [35] assesses the functional capacity of the care recipient in 8 domains of functioning. Scores total from 0 to 8, with higher scores indicating greater functional independence. The NPS inventory questionnaire [36] measures both the presence and severity of the care recipient’s NPS and the caregiver’s distress related to NPSs. Caregivers were also asked to complete measures related to their own well-being. These included the Beck Depression Inventory-II [37], a 21-item scale to assess the severity of depressive symptoms, and the Beck Anxiety Inventory [38], a 21-item scale to assess the severity of anxiety. The revised Caregiver Self-Efficacy Scale [39] is a 15-item scale exploring caregiver self-efficacy in obtaining respite, responding to patients’ disruptive behaviors, and controlling upsetting thoughts. The positive aspects of caregiving (PAC) questionnaire [40] consists of nine items that load onto two factors, self-affirmation and outlook on life, and assesses positive reflections on caregiving. The Relative Stress Scale [41] consists of 15 items and can be divided into 3 subgroups of emotional distress, social distress, and negative feelings. The meaning in life questionnaire [42] is a 10-item self-report assessment designed to measure the presence of and search for meaning in life.

At immediate follow-up only (ie, immediately after completing the TeleCARE intervention), participants also completed the client satisfaction questionnaire (CSQ-8) [43] to capture the acceptability of the intervention.

### Data Analysis

The primary objective of this pilot study was to assess the acceptability and feasibility of TeleCARE. Descriptive statistics were used because the goal was to explore trends rather than test hypotheses, as appropriate for the pilot stage of the current project. As such, we explored numerical trends and effect sizes of outcome variables. Descriptive statistics are reported to characterize the sample and summary scores for each measurement time point (baseline, post-treatment, and a 3-mo follow-up). Cohen *d* effect sizes were calculated to compare changes from baseline to post-treatment and from baseline to 3-month follow-up. All statistical analyses were conducted using SAS software (version 9.4; SAS Institute Inc). Given the early stage of this investigation, data from the TeleCARE test group were excluded; analyses only included participants in groups 2 to 6 who completed the final format of the TeleCARE intervention (n=17). An intent-to-treat approach was not used for this preliminary feasibility investigation.

## Results

### Participant Characteristics

Participants included 17 White caregivers (100%), who were predominantly female (n=13, 76%) and spouses (n=14, 82%), with a mean age of 71.8 (SD 12.58) years. All caregivers lived with the care recipient (n=17, 100%), and nearly all caregivers (n=16, 94%) reported providing the majority of care. Care recipients had a mean age of 77.7 (SD 4.84) years, were White (n=17, 100%), male (n=15, 88%), and had a mean clinical dementia rating sum of boxes score of 10.8, indicative of moderate dementia severity [44]. See Table 2.

**Table 2.** Baseline participant characteristics (N=17).

Characteristic	Participants
CR^a^ age (year), mean (SD)	77.7 (4.84)
CR sex (male), n (%)	15 (88)
CR race (White), n (%)	17 (100)
CG^b^ age (year), mean (SD)	71.8 (12.58)
CG sex (female), n (%)	13 (76)
CG race (White), n (%)	17 (100)
CG education, n (%)	
High school degree	1 (6)
Some college	8 (47)
College degree	7 (41)
Graduate degree	1 (6)
Household income (US $), n (%)	
0	1 (6)
1-24,999	2 (13)
25,000-49,999	2 (13)
50,000-74,999	7 (44)
75,000-99,999	3 (19)
100,000-149,999	0 (0)
150,000-199,999	1 (6)
Cohabitation, n (%)	
No	0 (0)
Yes	17 (100)
Relationship, n (%)	
Spouse/partner	14 (82)
Adult child	3 (18)
Percentage of caregiving, n (%)	
0%-25%	0 (0)
26%-50%	1 (6)
51%-75%	0 (0)
76%-100%	16 (94)
CDR^c^ sum of boxes, mean (SD)	10.8 (3.33)
IADL^d^, mean (SD)	1.9 (1.6)
NPI^e^, mean (SD)	
Total	6.9 (1.65)
Severity	15.5 (5.08)
Distress	26.8 (10.24)

a CR: care recipient.

b CG: caregiver.

c CDR: clinical dementia rating.

d IADL: Instrumental Activities of Daily Living.

e NPI: neuropsychiatric inventory.

### Feasibility Results: Recruitment, Enrollment, Attendance, and Retention

**Figure 1.**
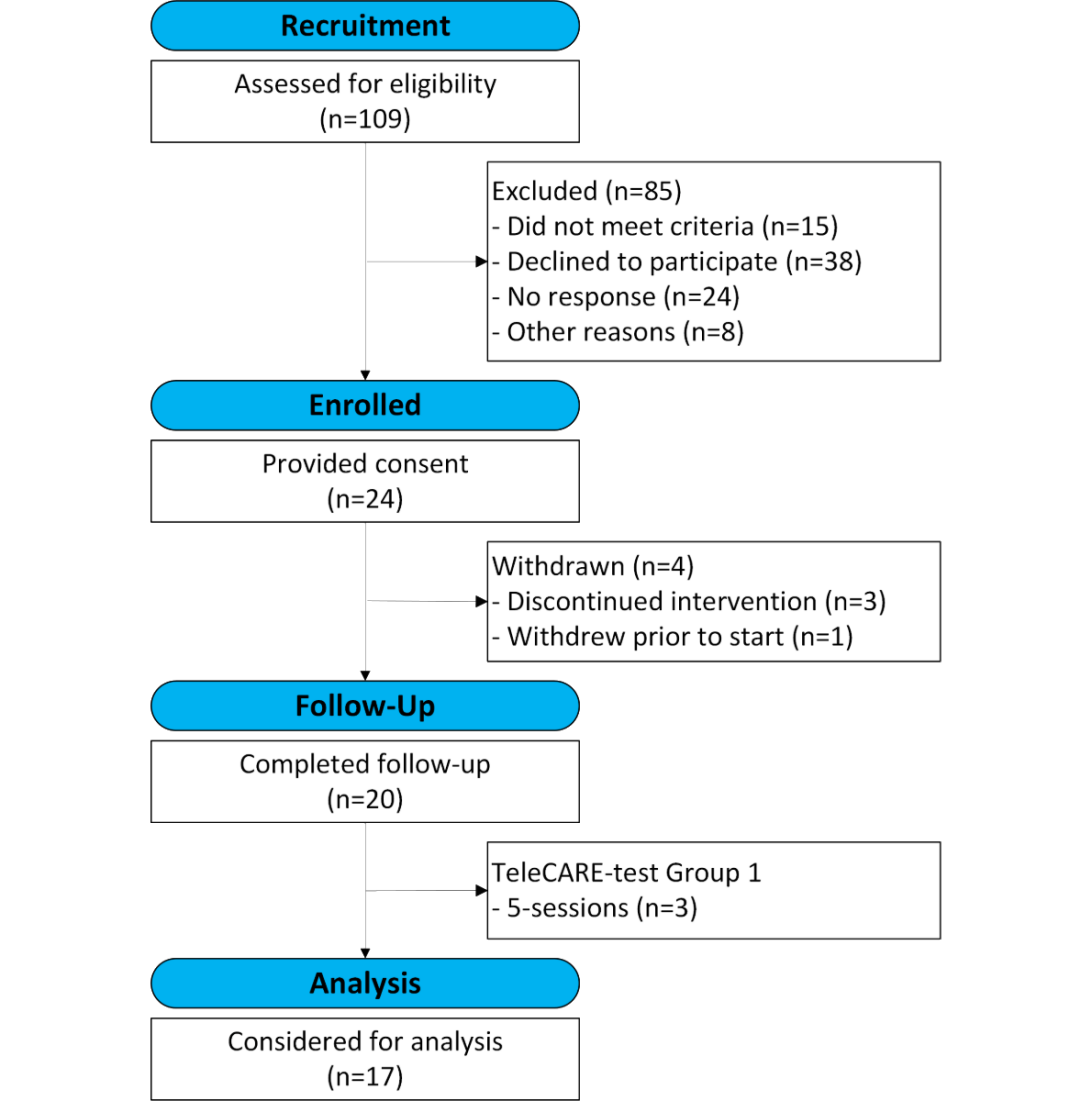
CONSORT (Consolidated Standards of Reporting Trials) flow diagram.

Of the 109 caregivers contacted for recruitment, 24 did not respond, 15 did not meet eligibility criteria, 38 declined participation, and 8 had other reasons (Figure 1). Among those who declined (38/109, 35%), the most common reason was that they felt that the intervention was not needed (20/38, 53%). The second most common reason was lack of time (11/38, 29%). The remaining provided no reason (3/38, 8%), said they were not interested (3/38, 8%), or reported a technological barrier (1/38, 3%). Of those that gave other responses (8/109, 7%), 5 said that their loved one was deceased when we contacted them, 2 had been admitted to long-term care, and 1 had recently been diagnosed with cancer. Overall, 24 caregivers (24/109, 22%) were enrolled in the study, with 4 in the TeleCARE-test group 1 and 20 in intervention groups 2 to 6. Across groups 1 to 6, 20 caregivers (20/24, 83%) completed the study. Of the 4 noncompleters, 1 was from the TeleCARE-test group 1, and 3 were from groups 2 to 6. Two of 4 noncompleters withdrew because they disliked the group setting. The remaining 2 noncompleters had medical issues arise that prevented continued participation (1 during the intervention and 1 prior to the start of the intervention but after study enrollment). Because the TeleCARE test group 1 was treated as a test group and subsequent changes were made to the intervention after their feedback, completers from TeleCARE test group 1 (3/4) were not included when reporting intervention attendance or exploratory quantitative analysis presented below. Of those within groups 2 to 6 (n=17), 10 attended all 7 sessions (10/17, 59%), 5 attended 6 of 7 sessions (5/17, 29%), and 2 attended 5 of 7 sessions (2/17, 12%). Occasionally, a participant arrived late, most commonly due to technological difficulties.

### Feasibility Results: Technology Support

**Figure 2.**
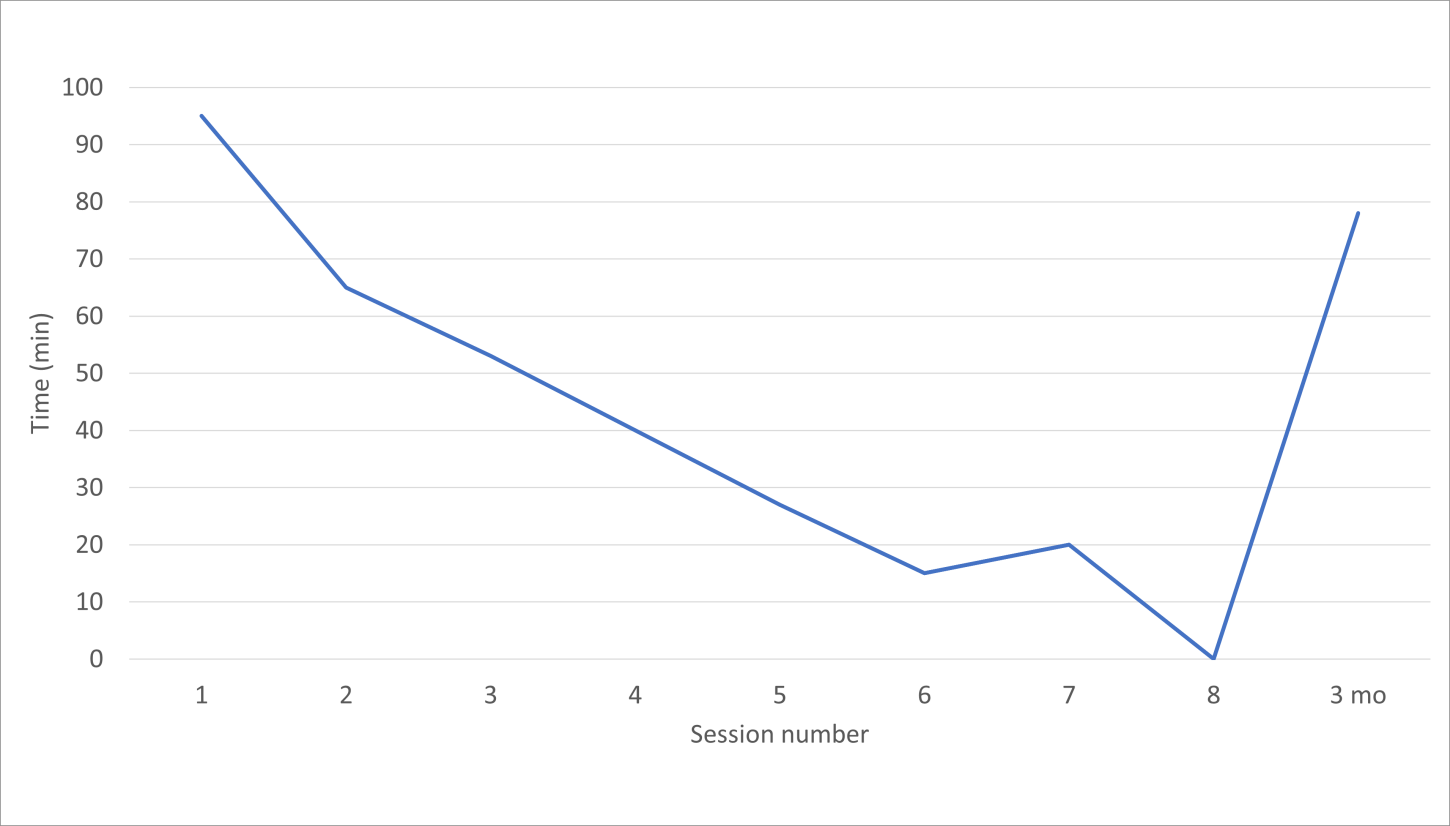
Total duration of technology assistance given before each session for all participants in groups 2 to 6.

To assess the acceptability and feasibility of the telehealth adaptation, data were collected to better understand participant experiences related to the use of technology. Overall, 11 of 17 participants (65%) required some degree of technology support. As mentioned above, a study staff member was assigned to provide personalized technology assistance as needed. Examples of when assistance was needed included helping participants turn on the power if a tablet was provided, access their email and click the video-meeting link, retrieve and/or reset their password, turn on their camera, use the mute and unmute function, and use the raise hand feature. One study participant had no prior experience with technology and needed assistance at all levels, from setting up an email account to using the device. The largest amount of technological assistance was required at session 1, dropping considerably by session 2. Participants needed less support with each passing session, although some continued support remained necessary at almost all time points. Participants reported feeling a sense of accomplishment and increased comfort with technology at the conclusion of the study. At the 3-month follow-up visit, 3 of 17 participants (18%) required assistance with technology, which was an increase compared with session 7 of the intervention. This finding suggests that most study participants were able to retain the taught skills, even after 3 months, while a minority of participants experienced a decay in skills. Figure 2 provides a visual depiction of the amount of time spent providing technological assistance across all sessions for intervention groups 2 to 6.

### Intervention Acceptability

Smith et al [45] proposed the following guidelines for the interpretation of overall CSQ-8 scores into 4 levels of client satisfaction: “poor” (scores 8‐13), “fair” (scores 14‐19), “good” (scores 20‐25) and “excellent” (scores 26‐32). The mean CSQ-8 score was 22.3 (SD 1.08), suggesting good intervention acceptance.

### Outcome Trends

As mentioned previously, this study was not adequately powered to detect statistical significance, as appropriate for the pilot stage, and the focus was on identifying numerical trends and examining effect sizes. Table 3 provides the mean scores of measures completed at every time point (n=17; also see Table S1 in Multimedia Appendix 1). Between baseline and immediate post-treatment time points, caregivers reported a decline in the care recipient’s ability to complete IADLs. There was no substantial change in the number of NPS reported by the caregiver, though there was a trend towards a decrease in the reported severity of NPS (Cohen *d*=0.16). Regarding caregiver psychological well-being, trends between baseline and post-treatment time points included a decrease in depression and anxiety symptoms and an increase in PAC and meaning and purpose. In fact, the largest effect between baseline and post-treatment was a decrease in anxiety. Participants reported little change in relative stress. Interestingly, despite improvements in psychological well-being, participants reported a decrease in caregiving self-efficacy between baseline and post-treatment, with the decrease in self-efficacy having the second largest effect size of the outcome measurements. Except for a statistically significant decrease in caregiver self-efficacy (*t*_16_=2.17, *P*=.045, Cohen *d*=0.21), no outcomes were statistically significant.

**Table 3.** Changes in clinical and psychological measures.

Measure	Baseline, mean (min-max)	Posttreatment, mean (min-max)	3-month follow-up, mean (min-max)
IADL^a^	1.9 (0‐5)	1.6 (0‐4)	1.5 (0‐3)
NPI^b^			
Total	6.9 (4-10)	6.8 (2-11)	7.1 (3-10)
Severity	15.5 (6-28)	14.5 (5-28)	15.7 (5-28)
Distress	26.8 (10-46)	25.7 (9-53)	25.8 (9-45)
BDI^c^	8.8 (1-33)	7.6 (0‐30)	8.5 (0‐26)
BAI^d^	8.3 (0‐25)	6.6 (0‐29)	9.6 (0‐40)
PAC^e^	34.6 (26-45)	35.8 (26-45)	34.4 (23-45)
RSS^f^	25.9 (9-47)	26.1 (11-42)	25.2 (9-45)
CSES^g^	1347.8 (880‐1820)	1280 (730‐1840)	1329.4 (900‐1840)
MPL^h^	37.9 (17-57)	39.3 (23-62)	39.8 (27-55)

a IADL: Instrumental Activities of Daily Living.

b NPI: neuropsychiatric inventory.

c BDI: Beck Depression Inventory.

d BAI: Beck Anxiety Inventory.

e PAC: positive aspects of caregiving.

f RSS: Relatives’ Stress Scale.

g CSES: Caregiver Self-Efficacy Scale.

h MPL: meaning and purpose in life.

Between baseline and 3-month follow-up, participants reported further decline in IADLs for the care recipient and an increase in NPS. In addition, most improvements reported between baseline and immediate post-treatment were lost by the 3-month follow-up. Participant meaning and purpose in life was the only improvement maintained from post-treatment to the 3-month follow-up. None of the changes between baseline and the 3-month follow-up visits were statistically significant.

## Discussion

### Feasibility and Acceptability

In this pilot study, we were able to recruit dementia caregivers and engage them in a telehealth adaptation of CARE (TeleCARE). Twenty caregivers completed TeleCARE, including 3 from an initial test group that informed intervention modifications, leaving 17 caregivers in the final intervention group. Caregivers were predominantly older female spouses of the care recipient. Most enrolled caregivers attended all sessions, suggesting that caregivers who engaged in the program stayed engaged throughout. Likewise, satisfaction with the intervention was good. Preliminary quantitative trends suggested immediate postintervention improvements in caregiver outcomes. Overall, TeleCARE was deemed feasible and acceptable, with preliminary quantitative results suggesting that TeleCARE has the potential for a larger-scale study. Future studies of TeleCARE could harness digital recruitment methods for scalability.

Importantly, intervention adaptations had to be made to successfully implement TeleCARE, including both intervention telehealth-specific modifications and the provision of broad technology support. Related to the modifications, previous research emphasizes the importance of safety and risk management, participant privacy, and platform etiquette in telehealth contexts. Telehealth inherently presents unique challenges compared to in-person care, including limited immediate clinical support and barriers to emergency response due to geographic variability and licensure restrictions [46-48]. Presession safety protocols, such as verifying participant location and emergency contacts, are increasingly recommended to mitigate risks during psychological or medical crises [47]. In addition to safety considerations, given evidence that clinical outcomes in telehealth are often comparable to in-person care, researchers emphasize the importance of replicating in-person standards through structured telehealth delivery [49], such as minimizing distractions, limiting movement, and encouraging stationary participation. Privacy concerns also remain central, with recommendations for participants to join from secure, confidential environments to protect sensitive information and foster psychological safety [48,50].

Compared with younger counterparts, older adults tend to have lower digital literacy, which presents a barrier to engaging in telehealth services [51]. Even after adding an initial (before the start of the intervention) technology tutorial session, the majority of study participants (11/17, 65%) required technological support, and though less support was needed after each passing session, some participants required ongoing support throughout. Digital literacy training programs for older adults have emerged in response to the recognized need to improve access to technology-based services [52-54]. Many studies have found that older adults want to engage with technology and not feel left behind as digital opportunities increase [55]. At study completion, participants reported feeling a sense of accomplishment related to learning to use the necessary technology and increased comfort with technology, highlighting the ability of older adults to engage with technology with individualized and ongoing support.

It is worth noting that half of the caregivers who declined to join the group stated that they did so due to a lack of time, despite the program being less than a 2-month, 90-minute weekly commitment offered via telehealth, thereby minimizing the time needed to travel. Dementia caregivers consistently identify time constraints as a major barrier to participating in interventions [56-58]. Many caregivers report prioritizing the needs of the care recipient over their own and neglecting self-care, thereby reducing opportunities to participate in supportive services [59-61]. Recent research suggests that brief, flexible, telehealth-based interventions may help boost caregiver engagement [62]. The current findings suggest that offering interventions via telehealth may be only one small step toward improving intervention engagement and that brevity and flexibility may play a bigger role in helping to engage busy caregivers. In addition, finding innovative ways to increase respite time for caregivers so they can engage in supportive services offers promise. For example, Iacob and colleagues [63] demonstrated that an online scheduling and planning program with virtual coaching and education helped increase caregiver respite [63].

### Outcome Trends

Although the study was not powered to statistically test changes between pre- and posttreatment, quantitative results suggested trends toward a decrease in NPS severity in care recipients, a decrease in caregiver depression and anxiety, and an increase in caregiver-expressed PAC and meaning and purpose in life after intervention completion. However, most improvements were not sustained by the 3-month follow-up. Also of note is the reduction in caregiving self-efficacy following intervention engagement, which may be unexpected. However, when learning new skills, individuals often experience initial failures early in the learning process. Although these failures are part of the growth necessary to learn new skills, failures can lead to temporary reductions in self-efficacy [64]. It is possible that caregivers in TeleCARE experienced reduced self-efficacy related to their initial unsuccessful efforts applying new skills. Caregivers may also have attributed their successes largely to the presence of the interventionist, causing self-efficacy to decline postintervention when transitioning to applying skills independently. In addition, skills may decay between the conclusion of an intervention and longer-term follow-up (in the current study, 3 mo), which can lead to further reductions in self-efficacy [65,66]. Future studies aimed at better understanding the conditions for gains in self-efficacy and sustainment of those gains are warranted. In addition, future trials of TeleCARE could incorporate theoretically driven self-efficacy enhancement techniques based on Bandura’s four identified self-efficacy enhancing methods (mastery experience, modeling, social persuasion, altering emotional/somatic states) [67], while retaining the basic intervention elements.

### Limitations and Future Directions

The major limitation of the current study is the homogeneous sample, which reduces generalizability. All of the participants in this study were white, mostly female, and primarily spouses of the individuals receiving care, with at least some college education. This fact limits the applicability of our findings to caregivers from diverse ethnic, cultural, and socioeconomic backgrounds.

Studies have found that the perception of caregiver differs for different cultures and, for those with more deprived socioeconomic disadvantage, knowledge about dementia may be low [68-70]. Intervention adaptations that take into account ethnic or cultural considerations and differences related to medical privacy, preferences for keeping the person with dementia at home, the role of religion, and the use of holistic or non-Western medicine approaches to care would be important to consider in future trials of TeleCARE [71-73]. TeleCARE may need to be adapted for caregivers with lower levels of education and lower income associated with limited or inadequate health literacy [74,75]. Of course, adapting the intervention for non-English speaking populations would improve reach. The original CARE intervention was successfully culturally and linguistically tailored for a Latino population and could serve as a basis for a TeleCARE adaptation in future trials [76]. Importantly, and aligned with the focus of telehealth adaptations, racial and ethnic minority caregivers and those with lower educational attainment are less likely to have access to technology (eg, stable internet connectivity, computer, smartphone) and the skills needed to engage with technology, and specific efforts to explore the use of telehealth for diverse cohorts of dementia caregivers is critical [77-80]. Building community partnerships for intervention adaptation, outreach, and recruitment can help strengthen future trials.

The predominantly female participants in the current intervention limit our ability to fully capture male caregivers’ experiences. Male caregivers may differ in their use of coping strategies, emotional responses, and support-seeking behaviors, with literature suggesting a greater tendency to use problem-focused strategies, seek help earlier, and prioritize having time for themselves, engaging in personal activities that may buffer caregiving stress [81,82]. As caregiving intensity increases, male caregivers may experience higher burden than their female counterparts, who often develop caregiving-related stress earlier in the process [81-83]. Thus, interventions that have predominantly female participants may differ significantly from a predominantly male group. The current study also included predominantly spousal caregivers. Adult child caregivers face distinct challenges, including the need to balance caregiving responsibilities with employment and parenting obligations [84,85]. Future research should continue exploring these subgroup differences to tailor interventions accordingly.

In summary, we successfully adapted an existing evidence-based, in-person, manualized dementia caregiver intervention (CARE) for synchronous video telehealth demonstrating good feasibility and acceptability. Successful adaptation required revising the manualized protocol to include procedures to ensure participant safety, etiquette instructions to improve group experience, and technological support to increase accessibility. These findings suggest that when attempting to transition an in-person program to video telehealth, these broad program-nonspecific adaptations should be considered. The important need for ongoing, available technological assistance was particularly salient.

## Supplementary material

10.2196/81256 Multimedia Appendix 1 Standard deviations and effect sizes of changes in clinical and psychological measures.

## Abbreviations

CONSORT: Consolidated Standards of Reporting Trials
CSQ-8: Client Satisfaction Questionnaire
IADL: Instrumental Activities of Daily Living
NPS: neuropsychiatric symptoms
PAC: positive aspects of caregiving
REACH: resources for enhancing Alzheimer’s caregiver health
REDCap: Research Electronic Data Capture
VA: Veterans Affairs
