# Supplementary material for: A Telehealth-Adapted Dementia Caregiver Skills Training Intervention (TeleCARE): Single-Arm Pre-Post Intervention Study
Source: JMIR Aging. 2026 Mar 12;9:e81256. doi: 10.2196/81256 (PMC12981637; doi:10.2196/81256)
Supplement: Multimedia Appendix 1 [file aging-v9-e81256-s001.docx]

| Table S1. Standard deviations and effect sizes of changes in clinical and psychological measures. | | | | | |
| --- | --- | --- | --- | --- | --- |
|  | Baseline | Post-treatment | | 3-month follow-up | |
|  | SD | SD | *d*^a^ | SD | *d* |
| IADL^b^ | 1.60 | 1.37 | 0.20 | 1.12 | 0.29 |
| NPI^c^  Total  Severity  Distress | 1.65  5.08  10.24 | 1.65  7.23  12.39 | 0.06  0.16  0.10 | 1.98  6.63  10.81 | 0.11  0.03  0.09 |
| BDI^d^ | 7.96 | 7.66 | 0.15 | 7.91 | 0.04 |
| BAI^e^ | 7.55 | 7.14 | 0.23 | 11.67 | 0.13 |
| PAC^f^ | 7.25 | 6.16 | 0.18 | 6.44 | 0.03 |
| RSS^g^ | 11.10 | 10.12 | 0.02 | 10.67 | 0.06 |
| CSES^h^ | 307.62 | 345.44 | 0.21 | 311.09 | 0.06 |
| MPL^i^ | 10.17 | 18.29 | 0.09 | 6.64 | 0.22 |
| ^a^Cohen *d* interpretation guidelines: 0.2=small effect, 0.5=medium effect, 0.8=large effect. | | | | | |

^b^IADL: instrumental activities of daily living.

^c^NPI: neuropsychiatric inventory.

^d^BDI: Beck depression inventory.

^e^BAI: Beck anxiety inventory.

^f^PAC: positive aspects of caregiving.

^g^RSS: Relatives’ Stress Scale.

^h^CSES: Caregiver Self-Efficacy Scale.

^i^MPL: meaning and purpose in life.
